# HMGB1 induction of clusterin creates a chemoresistant niche in human prostate tumor cells

**DOI:** 10.1038/srep15085

**Published:** 2015-10-15

**Authors:** Junmin Zhou, Xianghong Chen, Danielle L. Gilvary, Melba M. Tejera, Erika A. Eksioglu, Sheng Wei, Julie Y. Djeu

**Affiliations:** 1 Immunology Program, Moffitt Cancer Center, Tampa, FL 33612.

## Abstract

Development of chemoresistance, especially to docetaxel (DTX), is the primary barrier to the cure of castration-resistant prostate cancer but its mechanism is obscure. Here, we report a seminal crosstalk between dying and residual live tumor cells during treatment with DTX that can result in outgrowth of a chemoresistant population. Survival was due to the induction of secretory/cytoplasmic clusterin (sCLU), which is a potent anti-apoptotic protein known to bind and sequester Bax from mitochondria, to prevent caspase 3 activation. sCLU induction in live cells depended on HMGB1 release from dying cells. Supernatants from DTX-treated DU145 tumor cells, which were shown to contain HMGB1, effectively induced sCLU from newly-plated DU145 tumor cells and protected them from DTX toxicity. Addition of anti-HMBG1 to the supernatant or pretreatment of newly-plated DU145 tumor cells with anti-TLR4 or anti-RAGE markedly abrogated sCLU induction and protective effect of the supernatant. Mechanistically, HMGB1 activated NFκB to promote sCLU gene expression and prevented the translocation of activated Bax to mitochondria to block cell death. Importantly, multiple currently-used chemotherapeutic drugs could release HMGB1 from tumor cells. These results suggest that acquisition of chemoresistance may involve the HMGB1/TLR4-RAGE/sCLU pathway triggered by dying cells to provide survival advantage to remnant live tumor cells.

Resistance to anticancer agents is one of the primary impediments to effective cancer therapy and its resolution remains a pressing issue^1^. Intrinsic pathways already existent within the tumor cell as well as new pathways triggered during drug treatment may play a role in preventing cell death. Despite intense effort to unravel the intrinsic and extrinsic pathways that mediate chemoresistance, it is still unclear which survival process dominates, especially in cancer patients undergoing prolonged treatment with a specific therapeutic agent. Recent focus has established clusterin (CLU) as a key contributor to chemoresistance to anticancer agents. We have previously shown that sCLU, in its secretory/cytoplasmic form, is a potent anti-apoptotic factor and is found to be differentially overexpressed in docetaxel (DTX)-resistant human prostate tumor cells in comparison to their drug-sensitive counterparts^2^,^3^. More significantly it is upregulated in advanced stage and metastatic cancers encompassing prostate, renal, bladder, breast, ovarian, colon, cervical, pancreatic carcinoma, hepatocarcinoma as well as melanoma and lymphoma^4^,^5^,^6^,^7^,^8^,^9^,^10^,^11^. Furthermore, sCLU expression is documented to lead to broad-based resistance to other unrelated chemotherapeutic agents such as doxorubicin, cisplatin, etoposide, and camphothecin^12^,^13^. Resistance to targeted death-inducing molecules, Tumor Necrosis Factor, Fas and TRAIL, histone deacetylase inhibitors or DNA damaging agents can also be mediated by sCLU^14^,^15^,^16^,^17^,^18^. Long known as a stress-induced chaperone, its mechanism of action as a prosurvival protein was found to be linked to its ability to stabilize Ku70/Bax complexes in the cytoplasm, sequestering Bax from translocating to mitochondria to induce cytochrome c release, and thus preventing a caspase 3 cascade and resultant apoptosis^13^,^19^. In fact, blockade of sCLU by specific antisense probes or antibody can resensitize chemoresistant tumor cells to drug treatment, and targeting of sCLU is a potential new strategy being attempted in the clinic to overcome chemoresistance^20^,^21^,^22^. However, what triggers sCLU production in chemoresistant tumor cells remains unanswered.

In deciphering the cause of sCLU induction, we earlier found that it was essential for the tumor cells to be cultured in DTX in a full monolayer in order to develop drug resistance. Sparser distribution of tumor cells inevitably caused complete cell destruction by DTX while, in the full monolayer, remnant tumor cells were capable of surviving DTX treatment to grow out into drug-resistant cell lines. This phenomenon intrigued us to question if dying tumor cells may contribute to the survival of neighboring cells. Of the mediators released by dying cells, High Mobility Group Box 1 (HMGB1) is the most likely candidate. HMGB1 is a stress-related pleiotrophic protein that participates not only in inflammation but also in tissue remodeling to achieve wound repair^23^,^24^,^25^. It has highly versatile biological activities, depending on its location, post-translational modification and context of the cell. Originally identified as a nonhistone, chromatin-associated protein^26^, it is a conserved gene expressed in all eukaryotic cells^27^. In normal cell physiology, HMGB1 is predominantly a nuclear DNA-binding protein that stabilizes nucleosomes and facilitates the assembly of site-specific DNA-binding complexes to promote recombination as well as gene transcription^28^,^29^. Upon necrotic cell death or late apoptosis associated with secondary necrosis, however, it is passively released^30^. Outside the cell, HMGB1 has an entirely different role, becoming a danger-associated molecular pattern (DAMP) or alarmin to activate the innate immune system either alone or in conjunction with cytokines or bound DNA^31^,^32^,^33^,^34^. It is established to have multiple effects, with the capacity to induce cytokines^31^, chemotaxis^35^, cytoskeletal reorganization^36^, differentiation^35^, tissue repair and regeneration of numerous cell types including endothelial cells^37^, smooth muscle cells^36^ and myocardial cells^38^. Thus, HMGB1 has the capacity to alter the environment where it is released.

In this study, we discovered that HMGB1 controls yet another pathway, via sCLU induction to promote cell survival. Using prostate cancer as a focus, where acquired resistance to docetaxel (DTX) signifies a point of no return for patients, we demonstrate that DTX acts by a paracrine mechanism to promote tumor cell survival and outgrowth via a HMGB1/TLR4-RAGE/sCLU pathway.

## Results

**Figure 1:**
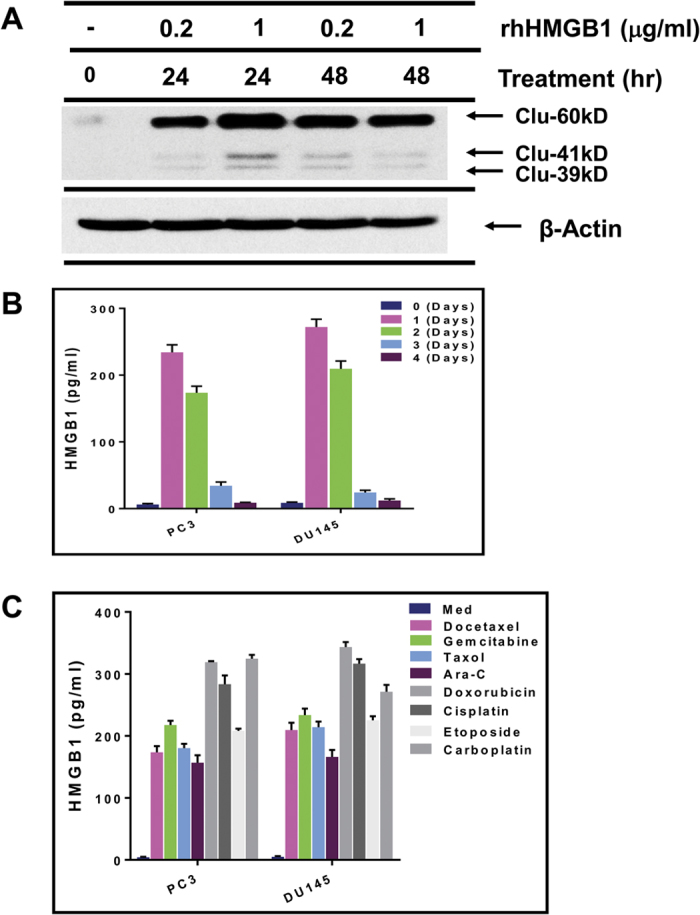
HMGB1 induces clusterin from tumor cells and is released by dying tumor cells. (**A**) DU145 prostate tumor cells, incubated with recombinant human HMGB1 (rhHMGB1) for 24–48 h, were lysed and analyzed by western blot for presence of clusterin, and for β-actin for equal loading. (**B**) DU145 tumor cells were treated with docetaxel (DTX) for 1–4 days and their supernatants were evaluated for HMGB1 by ELISA. (**C**) DU145 tumor cells were treated with the indicated chemotherapeutic agents for 24 h and the supernatants analyzed for HMGB1.

In initial experiments, we first sought to identify if recombinant human HMGB1 can induce sCLU from tumor cells. We chose to use DU145 and PC3 prostate tumor cells because our previous studies have documented that it expresses little or no sCLU, but this prosurvival protein can nevertheless be induced when the tumor cells acquire a chemoresistant phenotype^2^,^3^. The DU145 tumor cells were thus treated with 2 different doses of recombinant HMGB1 for 24–48 h and assessed for sCLU production. Western blot analysis with specific anti-CLU was performed on cell lysates in order to detect the uncleaved cytoplasmic 60 kD which has consistently been reported to confer survival advantages to tumor cells and is detected in most advanced stage cancers thus far tested^4^. The sCLU form, when matured and glycosylated for secretion, is made up of α and β chains of 41 kD and 39 kD, a small amount of which are also detected along with the 60 kD band^4^. Exposure to HMGB1 was found to induce significant levels of sCLU in DU145 tumor cells. At 0.2 ug/ml, HMGB1 requires 48 h to induce maximal levels of sCLU but, at 1 ug/ml, it rapidly induces maximal sCLU production by 24 h (Fig. 1A). Thus, HMGB1 is a potent stimulator of sCLU production in DU145 tumor cells. β-actin was used as a control for equal loading here and in all relevant subsequent experiments.

Given that recombinant HMGB1 can induce sCLU from tumor cells, we queried whether DTX could induce HMGB1 release. For this purpose, DU145 and PC3 prostate tumor cells were cultured with 55 nM of DTX, a dose previously determined by us to cause massive cell death, after which their supernatants were collected from day 0 to day 4 for ELISA analysis of HMGB1. HMGB1 could be found in the supernatants, peaking between day 1 and day 2, followed by a rapid decline (Fig. 1B). Additionally, drugs currently used in cancer patients, including gemcitabine, taxol, Ara-C, doxorubicin, cisplatin, etoposide, and carboplatin, used at a dose of twice the IC_50_ which caused necrosis and cell death, could readily induce HMGB1 release within 24 h of treatment (Fig. 1C). Thus, chemotherapeutic agents can universally cause tumor cell death, accompanied by HMGB1 release.

**Figure 2:**
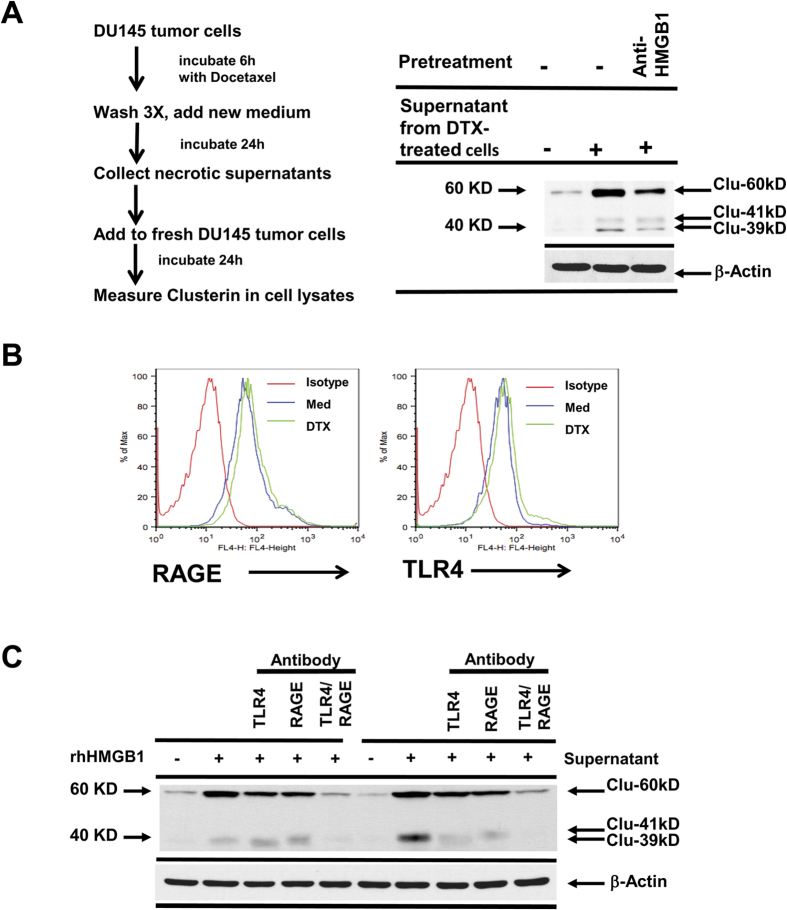
HMGB1 induces clusterin via TLR4 and RAGE. (**A**) DU145 tumor cells, pretreated with DTX for 6 h, were washed and then reincubated in fresh medium for another 24 h. Supernatants were collected and added to freshly-plated DU145 tumor cells for 24 h, either untreated or pretreated with anti-HMGB1 for 30 min. (**B**) Flow cytometric analysis of DU145 tumor cells treated with DTX for 24 h demonstrated no change for RAGE and TLR4 expression. (**C**) DU145 tumor cells, untreated or pretreated with anti-TLR4, anti-RAGE or both antibodies for 30 min, were exposed to recombinant HMGB1 or supernatants from DTX-pretreated tumor cells. After 24 h, the cells were lysed and analyzed for presence for clusterin by western blot.

In order to test our concept that HMGB1 from necrotic tumor cells can induce sCLU from live tumor cells, we next collected supernatants from DU145 tumor cells pretreated with DTX for 6 h and then washed free of DTX before reincubation for 24 h in drug-free medium (Fig. 2A). When this supernatant was added to freshly-plated DU145 tumor cells, it effectively raised cytoplasmic sCLU levels which could be markedly reduced by the presence of anti-HMGB1. Thus, DTX-treated tumor cell supernatants contain HMGB1 that can induce sCLU.

**Figure 3:**
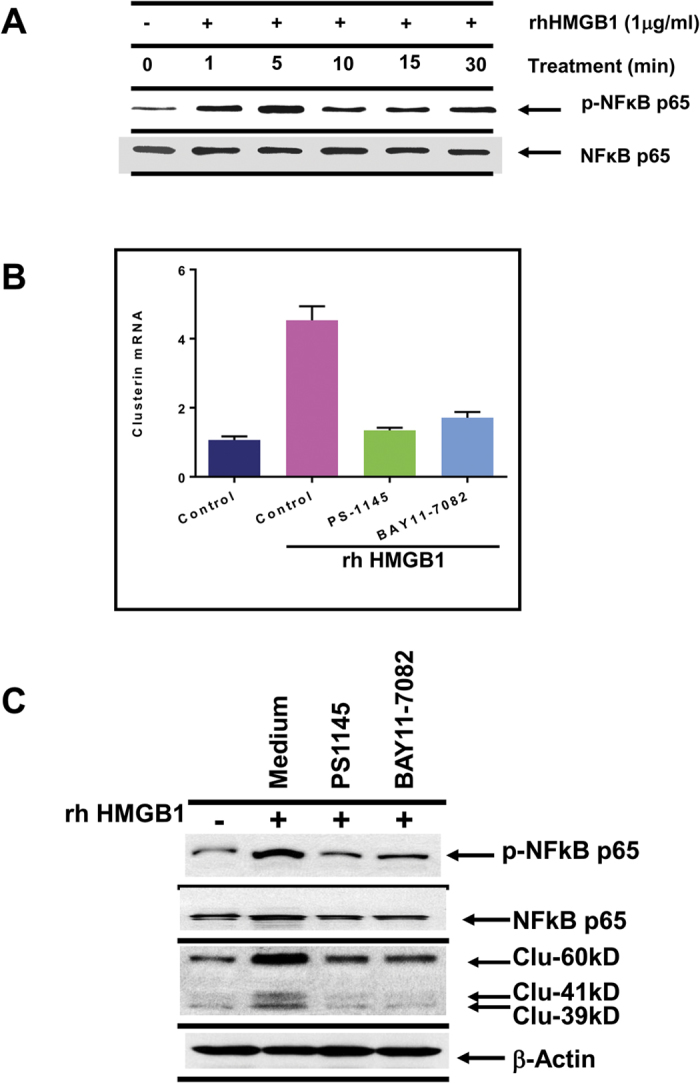
Down-regulation of clusterin expression after inhibition of NFκB-p65 phosphorylation. (**A**) Recombinant HMGB1, added to DU145 tumor cells, enhanced NFκB-p65 phosphorylation within 1–5 min, as analyzed by western blot. (**B,C**) DU145 tumor cells, pretreated with medium, 20 μM PS1145 or 10 μM BAY11-7082 for 1 h, were cultured with HMGB1 for another 24 h. Cells were then lysed for analysis of clusterin mRNA expression by Q-PCR or clusterin protein expression by western blot. Analysis of phosphorylated NFκB-p65 was included to check for effectiveness of the inhibitors.

The next step was to identify the receptor(s) expressed on DU145 tumor cells that could account for HMGB1 triggering of sCLU production. Both RAGE and TLR4, which are known receptors for HMGB1^31^,^39^, were detected by flow cytometry on DU145 tumor cells while DTX had no significant effects on the expression of these receptors (Fig. 2B). To identify which receptor was responsible for HMGB1 binding, we pretreated live DU145 tumor cells with anti-TLR4, anti-RAGE or the combination of both antibodies for 30 min prior to addition of recombinant HMGB1 (Fig. 2C). The data showed that recombinant HMGB1 induces high levels of sCLU and this effect was significantly blocked by anti-TLR4 or anti-RAGE alone, and the combination of both antibodies had a complete blockade, down to baseline levels. We reproduced the same results when supernatants from DTX-treated DU145 tumor cells were substituted for recombinant HMGB1 (Fig. 2C). Thus, both TLR4 and RAGE contribute to the response to HMGB1. Furthermore, HMGB1 could rapidly activate NFκB, a hallmark of TLR4/RAGE signaling, in DU145 tumor cells, as assessed by NFκBp65 phosphorylation (Fig. 3A). To assess the contribution of NFκB to sCLU expression, DU145 tumor cells were pretreated with NF-κB inhibitors, PS-1145 or BAY11-7082, for 1 h prior to incubation with HMGB1 for 24 h. Quantitative RT-PCR showed that PS1145 or BAY11-7082 inhibited mRNA expression of sCLU induced by HMGB1 (Fig. 3B). In addition, western blotting confirmed a marked reduction in sCLU protein in the same DU145 cells treated with the NF-κB inhibitors (Fig. 3C).

**Figure 4:**
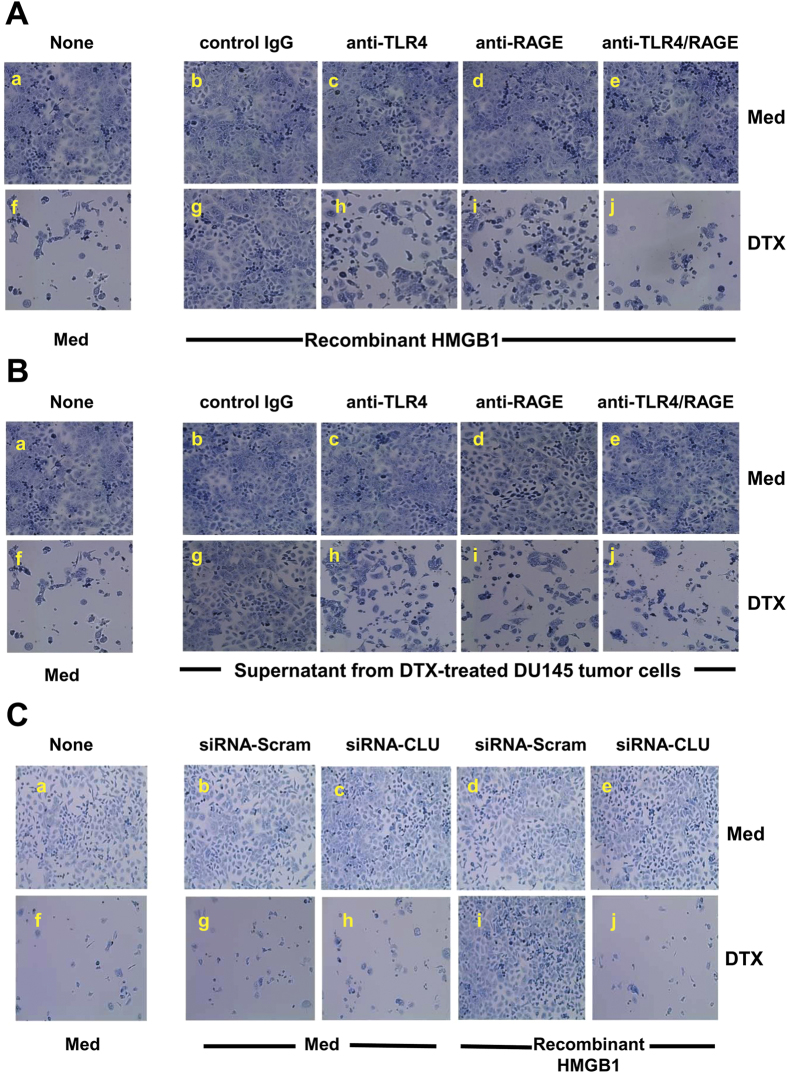
HMGB1 confers chemoresistance via TLR4 and RAGE. (**A**) DU145 tumor cells, untreated or pretreated with control IgG, anti-TLR4, anti-RAGE, or both antibodies for 30 min, were exposed to recombinant HMGB1 for 24 h. Then, DTX was added for another 48 h prior to staining with methylene blue for visualization of live tumor cells. (**B**) The same experiment was conducted with recombinant HMGB1 substituted for supernatants from DTX-treated tumor cells. (**C**) DU145 tumor cells pretreated with recombinant HMGB1 were transfected with antisense-scramble or antisense-clusterin for 24 h, and then cells were cultured in medium with or without DTX for another 48 h prior to staining with methylene blue.

It is important to verify that the induction of sCLU by HMGB1 can be translated into tumor survival and resistance to DTX treatment. For this purpose, DU145 tumor cells were exposed to recombinant HMGB1 and cultured in 55 nM DTX for 48 h (Fig. 4A). An aliquot of each cell group was pretreated with control IgG, anti-TLR4, anti-RAGE or anti-TLR4/RAGE to examine the receptors involved in induction of chemoresistance. It can be seen that DTX readily caused cell death, with few surviving cells (Fig. 4A–f) as compared to medium control (Fig. 4A–a). However, recombinant HMGB1-treated tumor cells (Fig. 4A–g) were completely protected and survived DTX treatment, as compared to tumor cells without HMGB1 (Fig. 4A–f). Thus, once tumor cells are exposed to HMGB1, chemotherapy fails to kill them. Anti-TLR4 (Fig. 4A–h) and anti-RAGE pretreated tumor cells (Fig. 4A–i), however, lost the protective ability of HMGB1, as compared to HMGB1-treated tumor cells with control IgG (Fig. 4A–g). Use of both anti-TLR4 and anti-RAGE had a more deleterious effect on tumor cells (Fig. 4A–j), suggesting that both receptors confer HMGB1-mediated drug resistance to DTX. Most importantly, the same results were obtained when necrotic supernatants were added to live DU145 tumor cells undergoing DTX treatment (Fig. 4B), supporting our notion that dying cells release HMGB1 in the supernatant that can bind tumor cells to confer drug resistance through sCLU induction. To prove that sCLU production caused drug resistance, we next utilized anti-sense CLU to block its expression in DU145 tumor cells prior to treatment with HMGB1. Scrambled siRNA control transfected DU145 tumor cells were protected by recombinant HMGB1 (Fig. 4C–i) with growth similar to medium control (Fig. 4C–a), even after treatment with DTX. However, HMGB1 could not protect anti-sense CLU-transfected DU145 tumor cells from DTX cytotoxicity (Fig. 4C–j), Thus, sCLU induction by HMGB1 mediates protection from DTX cytotoxity, resulting in drug resistance.

**Figure 5:**
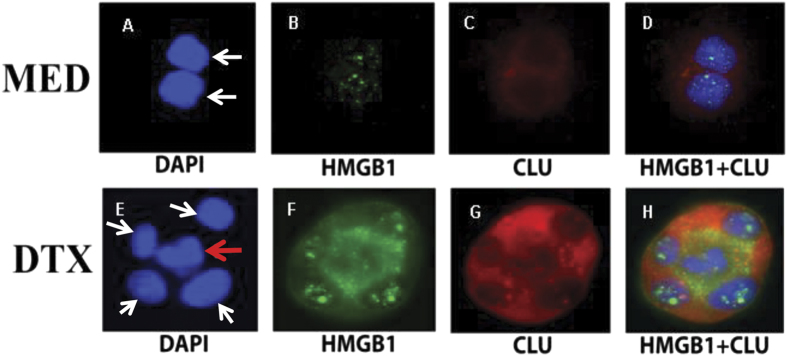
Dying tumor cells release HMGB1 which induces clusterin from neighboring live tumor cells. DU145 tumor cells were untreated or treated with DTX for 24 h and then dually stained for HMGB1 (green) and clusterin (red), as well as DAPI (blue) to visualize the nucleus. Live tumor cells (indicated by white arrows) in medium culture have intact round blue nucleus (**A**), intranuclear green speckled HMGB1 (**B**) and little cytoplasmic red clusterin (**C**). This separation of HMGB1 and clusterin is cleary seen in the merged picture (**D**). However, DTX treatment causes a change in the dynamics of the tumor cells. A DTX-treated dying tumor cell, indicated by a red arrow in the center of a cell cluster, has an irregular blue nuclei (**E**), and has released its green HMGB1 from the nucleus into the cytoplasm and surrounding medium (**F**). The dying cell is surrounded by 4 live tumor cells (white arrows), which still have intact nuclei with intranuclear speckled green HMGB1. More remarkably, these 4 live tumor cells now express high levels of cytoplasmic red clusterin. Thus, the HMGB1 released from the central dying tumor cells appear to have induced clusterin from the 4 neighboring tumor cells.

To prove that HMGB1 released from dying cells could bind a neighboring tumor cell to trigger sCLU and result in protection against DTX, we used immunohistochemistry to depict the molecules involved on a cell-by-cell basis. We cultured DU145 tumor cells in a full monolayer and then added 55 nM of DTX for 24 h, followed by dual immunostaining with anti-HMGB1 and anti-sCLU (Fig. 5). The purpose was to visualize the translocation of HMGB1 from the nucleus to the cytoplasm in dying cells and the induction of sCLU in residual live DU145 tumor cells in such cultures. Fig. 5A depicts 2 live cells (white arrows) with intact nuclei stained blue by DAPI (Fig. 5A). As expected, DU145 tumor cells, cultured in medium alone, contained HMGB1 (stained green) primarily in the nucleus (Fig. 5B), while sCLU (stained red) could hardly be detected in the cytoplasm (Fig. 5C), with no colocalization of HGMB1 with sCLU (Fig. 5D). After continuous culture in 55 nM of DTX for 24 h, most cells die and detach, leaving a few tiny clusters of cells. Examination of these clusters indicated that a dying cell can markedly affect its neighboring cell in both HMGB1 and sCLU content and location. A representative cluster, shown in Fig. 5E, demonstrates a dead cell (red arrow) in the center of a cluster of 4 live cells (white arrows). The nucleus of the dead cell has become irregular in shape and HMGB1 is almost completedly translocated into the cytoplasm and released (Fig. 5F). The four surrounding tumor cells, however, are alive, based on a round nuclear morphology with HMGB1 contained within the nucleus without any leakage to the cytoplasm (Fig. 5F), similar to those shown in the medium control (Fig. 5B). Contact with the dying cell, however, has markedly induced intense staining for sCLU (red) in the cytoplasm of these live cells (Fig. 5G), as compared to live cells untreated with DTX (Fig. 5C). Thus, we can depict a paracrine action of HMGB1 released from a dying cell on four live neighboring cells to induce sCLU, which can confer protection against DTX, facilitating the emergence of a chemoresistant population.

**Figure 6:**
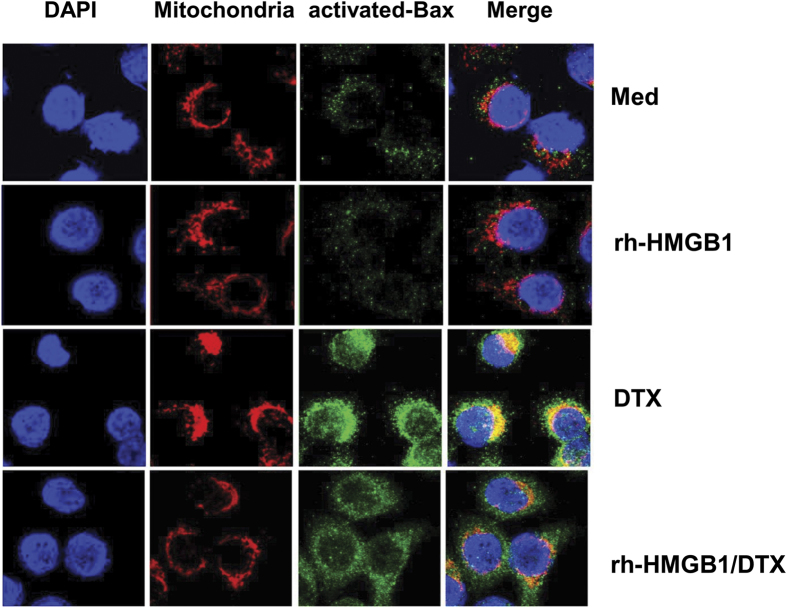
Clusterin blocks DTX-mediated apoptosis by sequestering Bax from mitochondria. DU145 tumor cells, pretreated with or without recombinant HMGB1 for 24 h, were treated with medium or DTX for 4 h. Mitochondria was visualized with MitoTracker Red, and activated Bax with 6A7 monoclonal antibody (green). Cells were examined by confocal microscopy (magnification, ×630) for colocalization of activated Bax with mitochondria. One representative image of three independent experiments is shown.

It has been reported that sCLU blocks apoptosis by interacting with activated Bax, thereby preventing activated Bax from translocating into the mitochondria, and exerting its proapoptotic activity^13^,^19^. We thus set out to examine if this pathway was utilized by recombinant HMGB1 to mediate cell survival and DTX resistance. To visualize activated Bax translocation to the mitochondria, we undertook a fluorimetric assessment of DU145 cells using monoclonal 6A7 antibody that specifically recognizes the activated, conformationally-altered form of Bax. Cell confocal imaging showed that DU145 tumor cells normally do not display activated Bax (Fig. 6, first row), but upon DTX treatment, gained high levels of activated Bax (green) that colocalized with mitochondria (red) (Fig. 6, third row). However, HMGB1-pretreated DU145 tumor cells showed less colocalization of activated Bax to mitochondria, when treated with DTX (Fig. 6, last row). These results indicate that HMGB1 can interfere with Bax mitochondrial translocation that can result in cell resistance to DTX cytotoxicity.

## Discussion

Acquisition of chemoresistance is a devastating barrier in cancer patients that often leads to clinical failure and poor survival. Acquired drug resistance can occur not only to clinically established therapeutic agents but also to novel targeted therapeutics. Our study brings new light to this critical issue by identifying a pathway in which dying cells play an active role in ensuring the survival of residual tumor cells that can form the seed of a chemoresistant population. First utilizing recombinant HMGB1, we show that it induces sCLU production from DU145 prostate tumor cells, which originally express little or no sCLU. Further we identified that recombinant HMGB1 works by binding to two of its known receptors, TLR4 and RAGE. To connect this HMGB1-TLR4/RAGE-sCLU pathway to chemoresistance, we were able to demonstrate that treatment of DU145 tumor cells with a cytotoxic dose of DTX caused the release of HMGB1 and the supernatant containing HMGB1 collected from DTX-treated tumor cells could in turn induce sCLU from live, untreated DU145 tumor cells. This action was due to HMGB1 binding to TLR4 or RAGE, as pretreatment of the supernatant with anti-HMGB1 or pretreatment of the live tumor cells with anti-TLR4/RAGE markedly inhibited sCLU induction. HMGB1 also triggered NFκB activation in DU145 cells to induce sCLU gene transcription and protein production.

The paracrine induction of sCLU in live cells by HMGB1 produced from dying cells could be visualized by immunohistochemistry. Tiny clusters of tumor cells that remain after DTX treatment were found to contain both dead and live cells. What was remarkable was the difference in location of HMGB1 in these cells. HMGB1 was primarily detected in the nucleus of live cells with little discernible levels of sCLU in the cytoplasm. However, HMGB1 can be seen to be translocated into the cytoplasm of dying cells and released into the surrounding environment. The live tumor cells in contact with the dying cell still retained HMGB1 in the nucleus. However, in response to the released HMGB1 from dead cells, they now express high levels of sCLU in their cytoplasm. In addition to visualizing the interaction of dead and live cells through HMGB1/sCLU induction, we also undertook a fluorescent approach to identify the mechanism of action of HMGB1 on live cells responding to DTX. We clearly demonstrated that DTX caused a translocation of activated Bax to the mitochondria, which results in apoptotic cell death. However, HMGB1 markedly inhibited this translocation, preventing Bax from initiating the apoptotic machinery.

We and others have established that sCLU is paramount in mediating survival in chemoresistant tumor cells^4^. This paracrine HMGB1-TLR2/4-sCLU pathway apparently may also be elicited by other current anti-cancer compounds, because gemcitabine, taxol, Ara-C, doxorubicin, cisplatin, etoposide and carboplatin could all readily induce HMGB1 release from dying tumor cells. Thus, it is reasonable to predict that this pathway may be universally induced in cancer patients that acquire resistance to any one of these drugs.

High expression of HMGB1 has been reported in numerous human maligancies, including breast, melanoma, gastric, colon, bladder, head and neck carcinoma^40^,^41^,^42^,^43^,^44^,^45^,^46^,^47^ and it is documented to be a key factor in a mouse model of inflammation-induced colon carcinogenesis^48^. The first receptor found to bind HMGB1 was RAGE which has been observed to be upregulated in numerous cancer types^39^,^45^, so is TLR4 which also binds HMGB1^35^,^49^. The binding site for TLR4 has been identified as HMGB1 89–108 residues with C106 being the critical amino acid in binding HMGB1^31^. This is in contrast to RAGE which binds a downstream domain at residues150–183^50^. There has been the notion introduced that RAGE may regulate chemotaxis, proliferation and differentiation in epithelial cells while TLR4 may dictate inflammatory cytokine release, particularly from macrophages and dendritic cells. Division of labor among these receptors do not appear to operate in prostate tumor cells, as they both could induce sCLU upon engagement of HMGB1 to drive cell survival and confer chemoresistance. As another mode of action, HMGB1 has recently been found to enhance beclin-1-associated autophagy to prevent stress-induced cell damage and apoptosis in osteosarcoma and leukemic cells, contributing to chemoresistance^51^,^52^. Interestingly, sCLU can also attenuate autophagosome biogenesis to promote cell survival^53^. Whether autophagy and sCLU synergize to drive chemoresistance or occurs separately depending on the tumor cell type has yet to be determined.

## Methods

### Generation of HMGB1-containing supernatants

The androgen-independent DU145 and PC3 prostate tumor cell lines (American Type Culture Collection (Rockville, MD) were maintained in RPMI 1640 medium containing 10% heat-inactivated FBS with 100 U/ml penicillin, 100-μg/ml streptomycin (complete medium). DU145 tumor cells, plated to full confluency, in tissue culture dishes, overnight at 37 °C, were treated with 55 nM of DTX (Moffitt Cancer Center Pharmacy) for 6 h at 37 °C, washed thrice with warm PBS, and then reincubated with complete medium just sufficient to cover the cells for 24 h at 37 °C. The supernatant was collected and spun free of cell debris, before being frozen in aliquots until use.

### Induction of clusterin

DU145 tumor cells, plated to confluency overnight in 6 well plates, were treated with 1.0 μg/ml recombinant human HMGB1 (R & D systems) or supernatants harvested from DTX-treated DU145 tumor cells. After 24 h or 48 h, the cells were collected and lysed by incubation at 4 °C for 30 min in 1% NP-40, 10 mM Tris, 140 mM NaCl, 0.1 mM PMSF, 10 mM iodoacetamide, 50 mM NaF, 1 mM EDTA, 1 mM sodium orthovanadate, 0.25% Na Deoxycholate 100 ul ALA, and 100 ul of phosphatase inhibitor cocktails I and II (Sigma). Whole Cell lysates were centrifuged at 12,000 g for 10 min to remove nuclei and cell debris. The protein concentration of the soluble extracts was determined by using the Bio-Rad (Bradford) protein assay. Separation of 50 μg of total protein was performed on 10% SDS-polyacrylamide gels, and transferred to a nitrocellulose membrane before immunoblotting with monoclonal anti- human clusterin (Upstate, 1:1000). Equal loading was checked by reblotting with anti-β-actin (Sigma-Aldrich, 1:5000) for equal loading.

### Analysis of TLR4 and RAGE

For TLR-4 staining, 5 × 10^5^ cells were incubated with anti-TLR4-APC (eBiosciences) for 30 min in 0.5% BSA in PBS on ice; for RAGE staining, cells were incubated with mouse anti-RAGE (Abcam) for 30 min on ice, washed with PBS and then incubated with an Alexa 647 anti-mouse secondary antibody (Invitrogen). The data were collected using a LSRII (BD Pharmingen, San Diego, CA), and the results were analyzed using Flowjo 6.3.4 software (TreeStar). After confirmation of their presence, DU145 cells were pretreated with 2.5 μg/ml anti-TLR4 (R&D Systems), 2.5 μg/ml anti-RAGE (R&D Systems), 2.5 μg/ml each of both antibodies, or control medium for 30 min in 6 well plates, before adding 1.0 μg/ml recombinant HMGB1 or supernatants harvested from DTX-treated DU145 tumor cells. After further 24 h incubation, cells were lysed and subjected to immunoblotting with anti-clusterin as described above.

### Methylene blue staining

DU145 tumor cells were treated first for 30 min at 37 °C with medium, control IgG, anti-TLR4, anti-RAGE or both antibodies in a 96 well plate. The cells were then treated with either recombinant HMGB1 or supernatants from DTX-treated tumor cells for 24 h at 37 °C. After additional culture in DTX-containing medium for 48 h at 37 °C, cells were washed twice with PBS, fixed with Methanol for 1 min, followed by staining with methylene blue for 1 min. The stained cells were washed twice with deionized water and allowed to dry overnight. Images were observed with a Leitz Orthoplan 2 microscope (Photometrics), and pictures were captured by a CCD camera with the Smart Capture Program (Vysis).

### Transfection

DU145 tumor cells were treated with recombinant HMGB1 for 24 h to induce clusterin, and then transfected with antisense-scramble or antisense-clusterin (Lipofectamine 2000; Invitrogen). Again at 24 h, cells were cultured in medium with or without DTX for another 48 h prior to staining with methylene blue.

### Immunohistochemistry

DU145 tumor cells, grown in covered wells of a microscope slide, were treated with 55 nM DTX for 24 h at 37 °C. Then, the cells were fixed and dually stained with anti-clusterin and anti-HMGB1. Briefly, slides were blocked with goat serum, followed by incubation with mouse anti-human clusterin (Upstate, 1:500) and rabbit anti-human HMGB1 (Cell Signaling, 1:200) overnight at 4 °C. Next day, slides were incubated with an Alexa 549 anti-mouse secondary antibody (Invitrogen, 1:1000) or Alexa 488 anti-rabbit secondary antibody (Invitrogen, 1:1000) for 30 min. The slides were mounted with Everbrite mounting medium with DAPI (Biotium). Slides were analyzed with an automated Zeiss Imager Z.1 upright microscope through a 63×/1.4 NA objective with DAPI, FITC, and Alexa 549 filters. Images were captured by using the AxioCam MRm CCD camera and Axiovision (Version 4.7; Carl Zeiss).

### Immunofluorescence staining

To detect Bax localization to mitochondria, DU145 tumor cells being grown on glass coverslips were incubated with 125 nM MitoTracker Red CMXRos for 30 min at 37 °C. These cells were fixed with ice-cold methanol for 15 min at −20 °C and permeabilized with 0.2% Triton X-100 in PBS for 10 min. Cells were blocked with goat serum for 30 min. Then cells were incubated with monoclonal anti-Bax (6A7, 1:200) for 60 min at room temperature. The immunocomplexes were incubated with Alexa 488 anti-mouse secondary antibody (1:1000) for 30 min, before coverslips were mounted onto slides using Everbrite mounting medium with DAPI. The signals and colocalization were detected using a confocal microscope (Carl Zeiss MicroImaging, Inc., Thornwood, NY).

### Enzyme-linked Immunosorbent Assay (ELISA)

DU145 cells or PC3 cells were treated with docetaxel (DTX) for 1–4 days or were treated with the indicated chemotherapeutic agents for 24 h. Supernatants were collected and analyzed for the presence of human HMGB1 by an ELISA kit (LS-F4038) from LifeSpan Biosciences.

### Quantitative RT-PCR

Cells were lysed in TRIzol (Invitrogen), and RNA was purified and converted to cDNA (iScript cDNA Synthesis kit; BioRad). Real-time PCR was performed with iScript Syber-Green Supermix (BioRad). Primer sequences are as follows: clusterin (F 5′TGCGGATGAAGGACCAGTGTGA -3′/R 5′-TTTCCTGGTCAACCTCTCAGCG - 3′); and GAPDH (F-5′-GAAGGTGAAGGTCGGAGT-3′/R-5′-GAAGATGGTGATGGGATTTC-3′). Experimental genes were normalized to GAPDH. Relative fold changes in expression were determined by using the comparative cycle threshold method (2−ΔΔCT).

## Additional Information

**How to cite this article**: Zhou, J. *et al.* HMGB1 induction of clusterin creates a chemoresistant niche in human prostate tumor cells. *Sci. Rep.* **5**, 15085; doi: 10.1038/srep15085 (2015).
